# Wait and trust: conservative management of bradyarrhythmias due to dengue infection: a case report

**DOI:** 10.1093/ehjcr/ytae696

**Published:** 2024-12-26

**Authors:** Maicol Cortez, Bryam López, Bryan Angulo, Milagros Palomino, Carlos Mancha

**Affiliations:** Department of Cardiology, Edgardo Rebagliati Martins National Hospital, Avenida Edgardo Rebagliati 490, Jesús María, Lima 15072, Perú; Department of Cardiology, Edgardo Rebagliati Martins National Hospital, Avenida Edgardo Rebagliati 490, Jesús María, Lima 15072, Perú; Department of Cardiology, Edgardo Rebagliati Martins National Hospital, Avenida Edgardo Rebagliati 490, Jesús María, Lima 15072, Perú; Department of Cardiology, Edgardo Rebagliati Martins National Hospital, Avenida Edgardo Rebagliati 490, Jesús María, Lima 15072, Perú; Department of Cardiology, Edgardo Rebagliati Martins National Hospital, Avenida Edgardo Rebagliati 490, Jesús María, Lima 15072, Perú

**Keywords:** Dengue, Heart conduction system, Arrhythmia, Sinus node dysfunction, Case report

## Abstract

**Background:**

This case report highlights the conduction disorder anomalies associated with dengue infection, particularly bradyarrhythmias due to dysfunction of the sinus node and atrioventricular node, which may require cardiac stimulation such as pacemaker implantation. This case emphasizes the importance of continuous monitoring and the use of additional diagnostic techniques to detect complications in a timely manner.

**Case summary:**

A 31-year-old male patient was admitted to our institution with symptoms of dyspnoea, orthopnoea, and severe bradycardia. During hospital admission, atrial fibrillation with a low ventricular response was evident. A 24-h Holter examination revealed additional electrical conduction abnormalities, including first-, second-, and third-degree atrioventricular block, 3.8 s pauses, and migrating atrial rhythm. Since the patient remained asymptomatic and did not present circulatory compromise, conservative management was chosen, with gradual recovery observed during the 30-day follow-up.

**Discussion:**

Dengue can significantly affect the cardiovascular system, presenting a variety of abnormalities in cardiac conduction. This case highlights electrical abnormalities and the importance of proper evaluation and management. It was decided to avoid temporary or permanent pacemaker implantation. This case underscores the need for continuous monitoring and the use of alternative diagnostic tools demonstrating that arrhythmias in this context can be successfully managed conservatively.

**Learning points**

- Understand the variety of arrhythmias and cardiac conduction abnormalities that can occur in dengue.
- Increase comprehension of the relationship between dengue and arrhythmias, highlighting the possibility of recovery without invasive intervention and the effectiveness of conservative management.

## Introduction

Dengue is a systemic viral disease with global presence, occurring in endemic and epidemic transmission cycles.^1^ The incidence of dengue has increased dramatically worldwide in recent decades. In 2023, the highest number of cases was recorded, affecting over 80 countries.^2^ The spread of this disease is due to various factors, such as ‘El Niño’ phenomenon and climate change, which increase temperatures, precipitation, and humidity; the weakness of health systems during the COVID-19 pandemic; political and financial instability; and large migratory movements.^3^ Although most dengue infections are asymptomatic or mild, it is estimated that between 1 and 5% of hospitalized patients develop complications, such as organ damage, haemorrhage, and plasma leakage.^4^ In severe cases, it can cause potentially life-threatening cardiovascular diseases, including dengue shock syndrome.^5^ Arrhythmias are common in dengue and involve multi-factorial pathogenesis.^6,7^ Electrocardiogram (ECG) abnormalities reported in dengue cases are usually transient and non-specific, including sinus bradycardia, atrioventricular block, and T-wave and ST-segment abnormalities.^8^

## Summary figure

**Figure:**
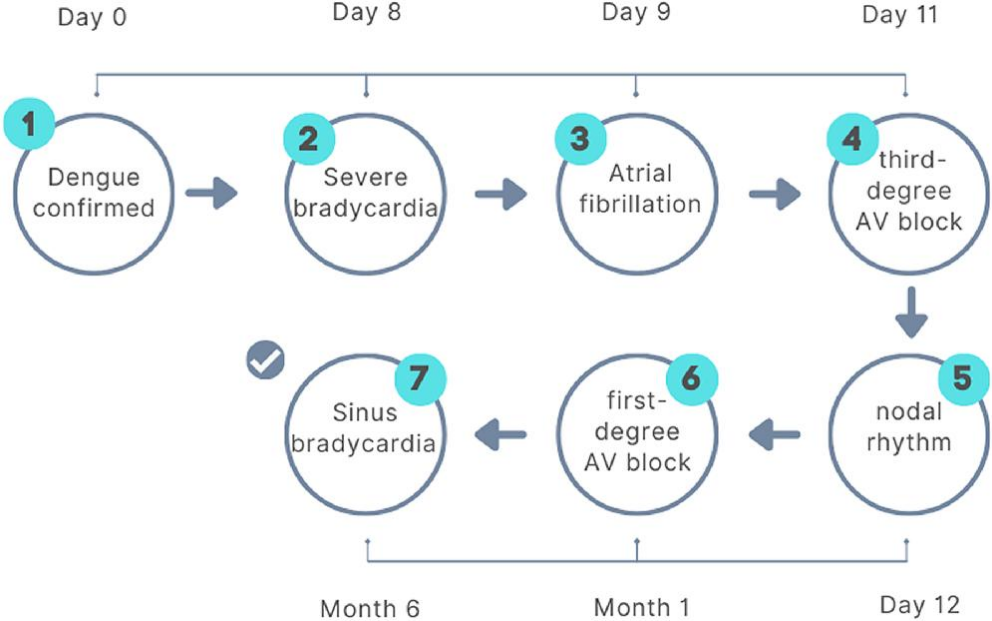


## Case presentation

A 31-year-old male with a history of illicit drug use (cocaine and marijuana), and no significant medical history, was initially hospitalized for dengue fever with warning signs, confirmed by Non-Structural Protein 1 (NS1) antigen testing. Treatment included hydration therapy and antipyretics. Four days after discharge, the patient developed dyspnoea on moderate exertion, orthopnoea, and bradycardia, prompting readmission. Subsequently, he was referred to our institution for evaluation and management of severe bradycardia.

**Figure 1:**
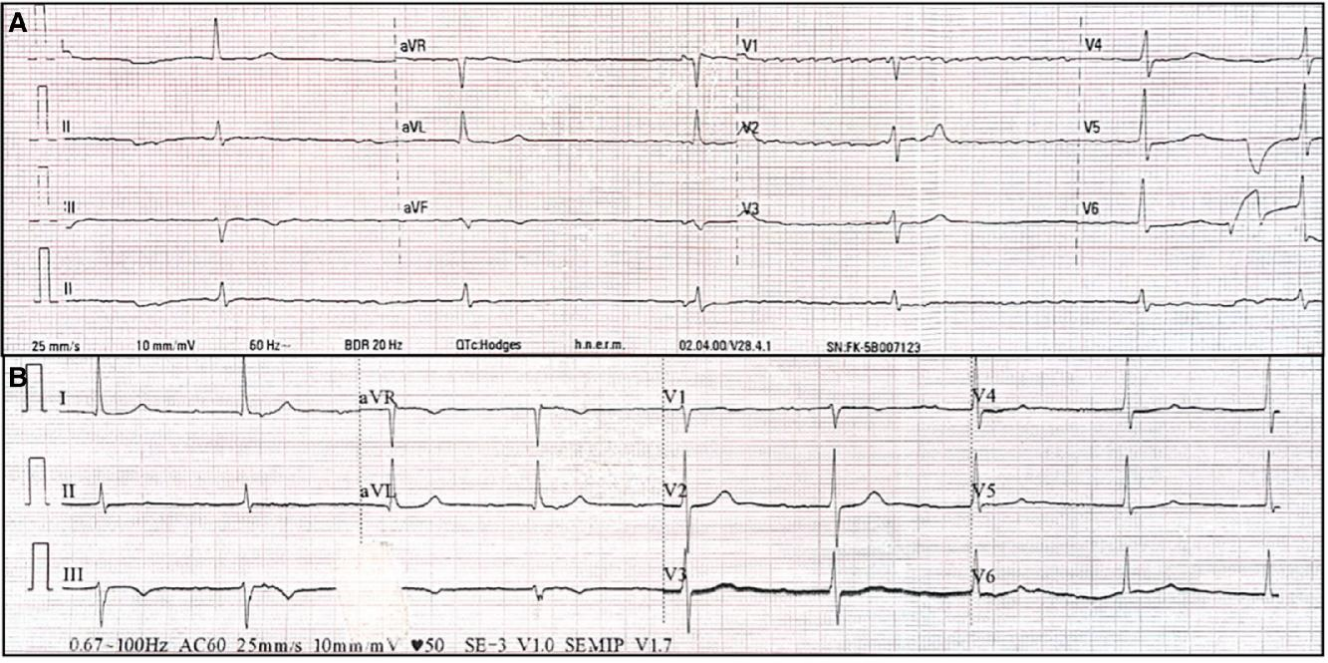
Electrocardiogram. (*A*) Atrial fibrillation rhythm with low ventricular response, 40 b.p.m. (*B*) Migratory atrial rhythm after electrical cardioversion.

**Figure 2:**
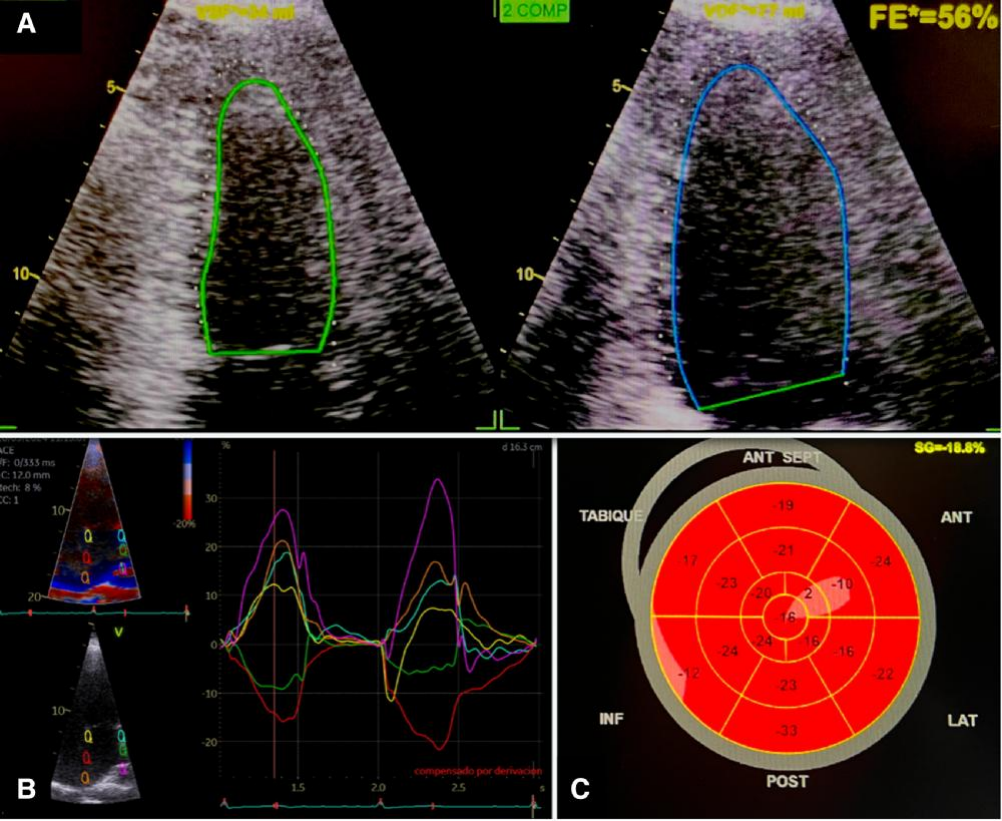
Transthoracic echocardiogram. (*A*) Preserved left ventricular ejection fraction. (*B*) Left atrial strain of 16%. (*C*) Left ventricular strain of −18.8%.

On admission, heart rate was 41 b.p.m., blood pressure 137/63 mmHg, and respiratory rate 13 breaths/min. Physical examination revealed bradycardic heart sounds, with otherwise unremarkable findings. Blood pressure remained stable throughout the episodes of third-degree atrioventricular (AV) block, with systolic pressures ranging between 110 and 130 mmHg and diastolic pressures between 70 and 80 mmHg, all within normal limits. Electrocardiography showed atrial fibrillation with a low ventricular response, along with T-wave abnormalities in Leads II, III, and aVF (*Figure 1A*). Metabolic studies during hospitalization were normal. Additional findings included Troponin-I at 24.3 pg/mL (reference range <17 pg/mL), Creatine Kinase-MB (CK-MB) at 4.3 ng/mL (reference range 0–6.3 ng/mL), and B-type Natriuretic Peptide (BNP) at 63 pg/mL (reference range 0–100 pg/mL). The initial transthoracic echocardiogram revealed mild left atrial dilation with a volume index of 37 mL/m² and preserved biventricular systolic function, including a left ventricular ejection fraction (LVEF) of 65% and tricuspid annular plane systolic excursion (TAPSE) of 24 mm. The left atrial strain was measured at 16% (*Figure 2*). Transoesophageal echocardiography did not detect thrombi in the left atrial appendage. Two electrical cardioversions were attempted at 200 J each, resulting in temporary atrial pacing with nodal escape rhythm (*Figure 1B*).

**Figure 3:**
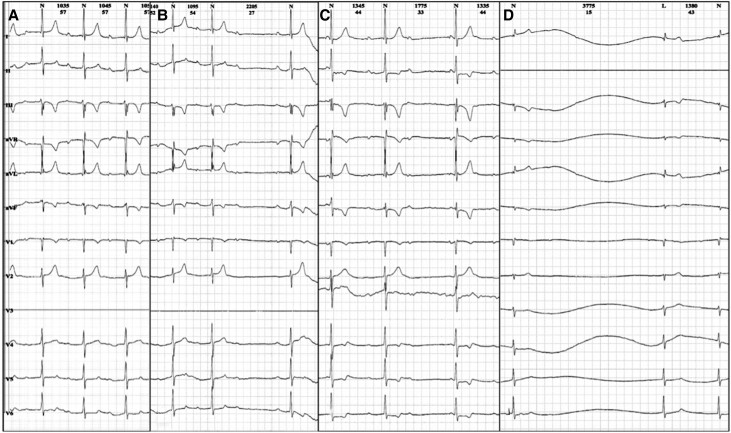
Holter. (*A*) First-degree atrioventricular block with a PR interval of 400 ms. (*B*) Second-degree Mobitz 2 atrioventricular block. (*C*) Third-degree atrioventricular block. (*D*) Sinus pause of 3.8 s with nodal escape.

**Figure 4:**
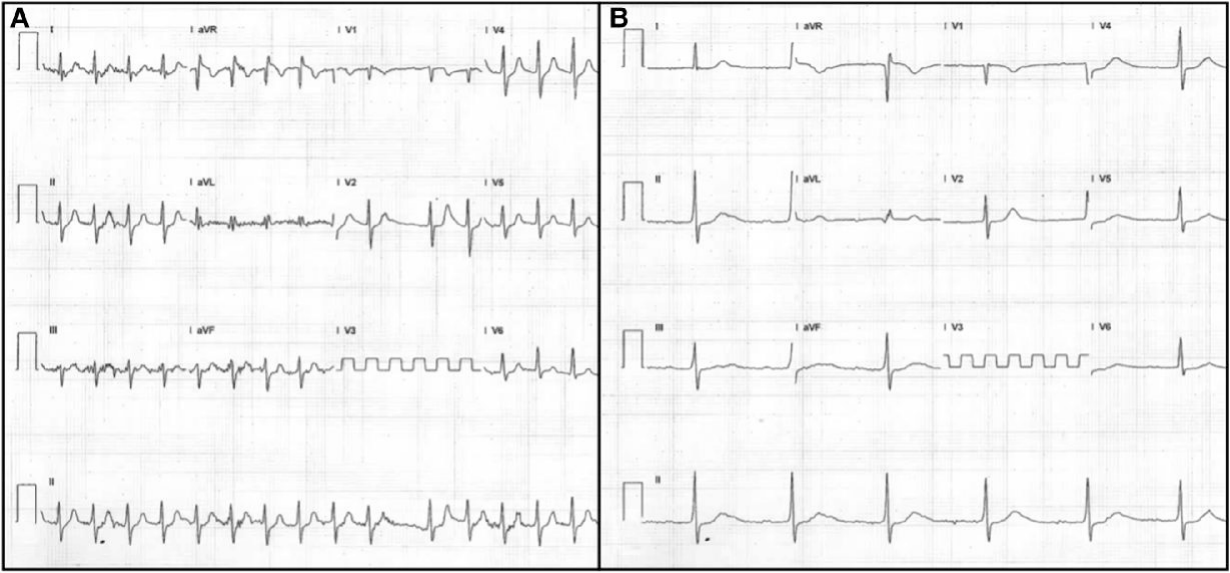
Exercise stress test. (*A*) Atrial fibrillation reaching up to 160 b.p.m. (*B*) Sinus rhythm during the recovery stage.

Holter monitoring revealed first-degree atrioventricular block with a PR interval of 400 ms, second-degree Mobitz 2 atrioventricular block, third-degree atrioventricular block, four sinus pauses, the longest being 3.8 s, and migratory atrial rhythm (*Figure 3*). Antiarrhythmic therapy was not considered during episodes of paroxysmal atrial fibrillation due to the presence of bradycardia and atrioventricular block. Anticoagulation was also not initiated because the CHA₂DS₂-VA score was 0 points, and there was no evidence of thrombi on echocardiography. The patient underwent an exercise stress test using the Bruce protocol to assess chronotropic response, revealing sinus rhythm, atrial fibrillation, and nodal rhythm, with a maximum heart rate of 160 b.p.m. (during atrial fibrillation) and achieving 10.2 METS (metabolic equivalents) with functional Class I (*Figure 4*). Coronary computed tomography angiography showed no evidence of atherosclerotic disease, normal coronary artery origins with left dominance, a calcium score of 0 AU, and CAD-RADS of 0.

**Figure 5:**
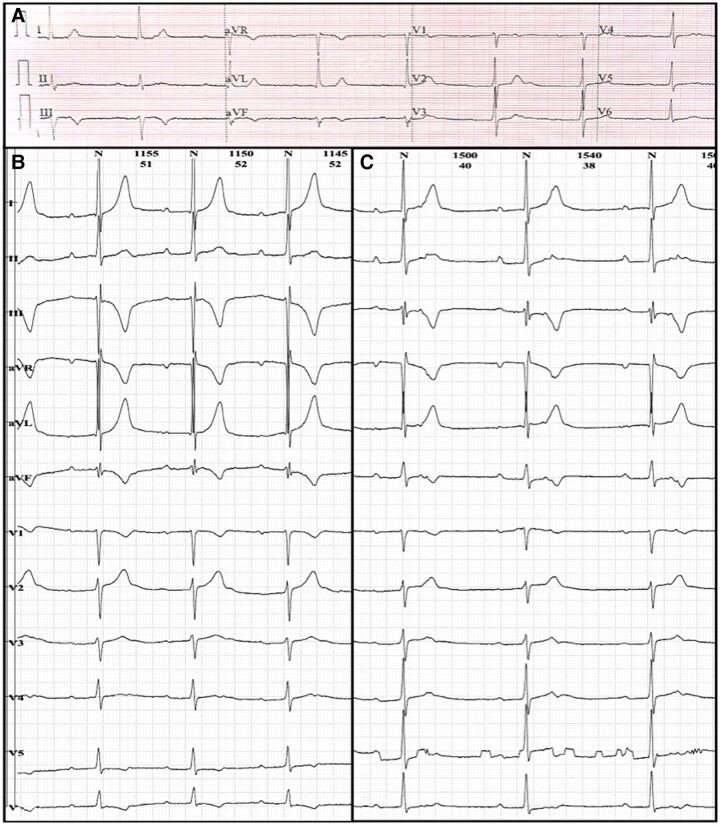
Follow-up after 30 days. (*A*) Electrocardiogram showing sinus rhythm with first-degree atrioventricular block (PR interval 320 ms). (*B*) Twenty-four-hour Holter monitoring demonstrating baseline sinus rhythm with first-degree atrioventricular block (PR interval 340 ms). (*C*) Minimum heart rate recorded at 39 b.p.m.

The patient was hospitalized for 7 days. Conservative management was chosen because the patient remained asymptomatic and showed no signs of circulatory compromise, leading to gradual recovery observed over a 30-day follow-up. The discharge criterion was based on a good chronotropic response during the exercise stress test. Holter monitoring at 30 days revealed only first-degree atrioventricular block with a minimum heart rate of 39 b.p.m., and at 6 months, the patient presented with sinus rhythm and a resting heart rate of 58 b.p.m. on ECG (*Figure 5*).

## Discussion

Dengue viral infection can significantly impact the cardiovascular system, affecting up to 46% of patients.^9^ Cardiac conduction system abnormalities and arrhythmias are highly variable in dengue, with common manifestations include sinus bradycardia (7% to 60%), sinus tachycardia (3.3% to 27%), first-degree (1.9% to 11%), second-degree (0.3% to 8.6%), and third-degree atrioventricular blocks (0.6%), as well as atrial fibrillation (0.9% to 3.75%), junctional rhythms (1.2% to 2.7%), and sinus pauses (2.9%).^6^

Arrhythmias are prevalent in dengue infection due to inflammation and cytokine release affecting myocardial cells, changes in ventricular dynamics, and increased myocardial oxygen demand.^10^ Additionally, low platelet counts increase the risk of haemorrhage around the sinoatrial or atrioventricular node, contributing to transient conduction abnormalities and arrhythmias.^11^

In the presented case, the patient exhibited multiple ECG abnormalities, including sinus node dysfunction, atrioventricular block, atrial fibrillation with slow ventricular response, and migratory atrial rhythm. These findings highlight the diverse cardiac conduction abnormalities associated with dengue. Despite these abnormalities, the patient remained asymptomatic during hospital monitoring. Moreover, a favourable heart rate response was observed during stress testing, achieving 85% of the maximum rate during atrial fibrillation. Consequently, it was decided not to pursue cardiac intervention.

Patient management involved rigorous monitoring and diagnostic investigations to explore secondary causes of cardiac injury. Coronary computed tomography angiography excluded coronary artery disease, while echocardiography provided insights into cardiac structure and function, confirming the absence of thrombi and structural abnormalities. Although literature reports describe cases involving temporary^12^ and permanent pacemaker implantation,^13^ recovery from sinus node dysfunction and atrioventricular block has been documented up to 5 months post-infection.^14,15^

This case demonstrates that arrhythmias, specifically bradycardia and atrioventricular block, associated with dengue can be successfully managed conservatively through continuous monitoring. The clinical condition of the patient, with no signs of circulatory compromise or haemodynamic instability, further supported the decision to avoid invasive interventions, such as pacemaker implantation. Long-term follow-up, including a repeat Holter and ECG at 6 months, will be essential to confirm recovery and detect potential recurrence of conduction abnormalities. This case highlights the effectiveness of a conservative management in dengue-related bradycardia and atrioventricular block.

## Data Availability

The data underlying this article are available to use for all readers.

## Lead author biography

Maicol Cortez Sandoval is a cardiologist at the National Hospital Edgardo Rebagliati Martins in Lima, Perú. He holds a Master’s in Clinical Cardiology from the Universidad Católica de Murcia and a Master’s in Cardiac Arrhythmology from Universidad Rey Juan Carlos. Additionally, he has specialized training in heart failure, coronary artery disease, and cardiac magnetic resonance imaging. Dr Cortez is dedicated to advancing cardiovascular care and research, with a particular focus on arrhythmias.

**Consent**: The authors confirm that written consent for the submission and publication of this case report, including images and associated text, has been obtained from the patient in accordance with COPE guidance.

**Funding:** The funding for the article was made by the main author.
